# Delivery-room and NICU admission temperatures in preterm infants <32 weeks at a tertiary centre in Saudi Arabia: associations with mortality and morbidity

**DOI:** 10.3389/fped.2025.1678128

**Published:** 2025-10-24

**Authors:** Kamal Ali, Mohanned Alrahili, Shaimaa Halabi, Mohamed Almahdi, Seham Alrsheedi, Amenah Alessa, Rana Almuqati, Talal Aljarbou, Mesaed Alsenani, Abdulrahman Mandurah, Faisal Alamer, Abdulrahman Altuwaym, Abdulaziz Homedi, Tarek Mohamed, Saif Alsaif

**Affiliations:** ^1^ Neonatal Intensive Care Department, King Abdulaziz Medical City-Riyadh, Ministry of National Guard Health Affairs, Riyadh, Saudi Arabia; ^2^ King Abdullah International Medical Research Center, Riyadh, Saudi Arabia; ^3^ College of Medicine, King Saud Bin Abdulaziz University for Health Sciences, Riyadh, Saudi Arabia

**Keywords:** preterm infants, hypothermia, thermal instability, NICU admission, neonatal outcomes

## Abstract

**Background:**

Despite implementation of thermal protection protocols, hypothermia remains common in preterm infants. The relative impact of hypothermia occurring in the delivery room (DR) vs. at NICU admission on neonatal outcomes remains insufficiently reported.

**Objective:**

To evaluate the association between hypothermia at two key time points—immediately after birth in the DR and upon admission to the NICU—and neonatal mortality and major morbidities in infants born at less than 32 weeks’ gestation.

**Methods:**

This retrospective cohort included inborn preterm infants <32 weeks’ gestation admitted to a tertiary NICU (January 2022–December 2024). Axillary temperatures were obtained after stabilization in the delivery room (DR) and again on NICU admission. Hypothermia was defined as <36.5 °C; infants were grouped by thermal status at each time point. Outcomes were in-hospital mortality and major morbidities. Associations were evaluated with multivariable logistic regression: morbidity models adjusted for gestational age, and mortality models adjusted for gestational age, major intraventricular hemorrhage (IVH), and sepsis; results are reported as adjusted odds ratios with 95% CIs.

**Results:**

Hypothermia occurred in 19% of infants in the DR and in 25% at NICU admission. The median temperature change between the DR and NICU was −0.1 °C [IQR: −0.2, 0.1], with a significant overall decline (*p* = 0.002). Mortality was higher in infants who were hypothermic in the DR (26.3% vs. 7.2%, *p* < 0.001) and at NICU admission (22.4% vs. 7.1%, *p* < 0.001). NICU hypothermia was independently associated with bronchopulmonary dysplasia (aOR: 1.89; 95% CI: 1.06–3.35), major IVH (aOR: 2.46; 95% CI: 1.05–5.75), and surgical necrotizing enterocolitis (aOR: 4.89; 95% CI: 1.48–16.17). Delivery-room hypothermia was associated with increased odds of BPD (aOR: 1.88; 95% CI: 1.02–3.46) but not with IVH or NEC. Infants hypothermic at both time points had the highest rates of mortality (25.9%) and BPD (59%).

**Conclusion:**

Hypothermia in the DR and at NICU admission is significantly associated with adverse outcomes, with the highest risk in infants hypothermic at both time points. These observational findings should be interpreted in light of potential selection bias, as smaller and sicker infants are more likely to be hypothermic, and highlight the need to prioritize thermoregulation from birth through admission.

## Introduction

Maintaining normothermia in the immediate postnatal period is a cornerstone of neonatal stabilization and resuscitation, particularly in preterm infants born before 32 weeks “gestation” (1). Neonatal hypothermia, defined as a core temperature below 36 °C, remains a globally recognized contributor to increased neonatal morbidity and mortality. Prior studies consistently link NICU admission temperature with adverse outcomes, including intraventricular hemorrhage (IVH), necrotizing enterocolitis (NEC), bronchopulmonary dysplasia (BPD), and mortality (2–6). Consequently, admission temperature is widely used as a quality indicator in delivery-room care and resuscitation guidelines.

Evidence on the clinical significance of the neonate's axillary temperature measured in the delivery room is limited. This is the first temperature taken after stabilization and before transport. How this value relates to the temperature at NICU admission is not well described. It is unclear whether changes during transport indicate early illness, reflect gaps in thermal care, or represent normal variation. It is also unclear whether differences between the two measurements are linked to adverse outcomes. These uncertainties motivated this study to evaluate DR and NICU axillary temperatures together and examine their associations with early neonatal outcomes.

This study evaluates the relationship between early postnatal thermal status and clinical outcomes in preterm infants <32 weeks' gestation. Our primary objective was to compare body temperature measured immediately after delivery-room stabilization with temperature on NICU admission. We also examined the association between time to NICU admission and temperature change. Finally, we assessed whether DR axillary temperature, NICU admission temperature, and their difference are independently associated with early neonatal mortality and major morbidities, using multivariable models adjusted for gestational age (morbidity models) and for gestational age, major IVH, and sepsis (mortality models).

## Methods

### Study design and setting

This retrospective cohort study was conducted at the Neonatal Intensive Care Unit (NICU) of King Abdulaziz Medical City (KAMC) in Riyadh, Saudi Arabia, a level III tertiary perinatal referral center. The study period spanned from January 2022 to December 2024. Ethical approval was granted by the King Abdullah International Medical Research Centre (KAIMRC) with an IRB number: (NRR25/018/6). Requirement for informed parental consent was waived due to the retrospective nature of the study and the use of de-identified data.

### Eligibility criteria

All inborn preterm infants delivered at less than 32 weeks of gestation during the study period were screened for inclusion. Infants were eligible if they had documented body temperature measurements at two specified timepoints: after stabilization in the delivery room (DR temperature) and at the time of NICU admission. Exclusion criteria included major congenital anomalies, outborn status, absence or delay in documentation of either temperature measurement, and neonatal death prior to NICU admission.

### Temperature measurement and thermal management

For all inborn infants, axillary temperature was measured with a calibrated digital thermometer [SureTemp Plus 692, Welch Allyn® (Hillrom), Skaneateles Falls, NY, USA] after initial stabilization in the delivery room (DR), typically within 5–10 min of birth, and again immediately on NICU admission before further interventions. Readings were recorded once unless implausible, in which case the measurement was repeated after probe repositioning. Hypothermia was defined as <36.5 °C and hyperthermia as >37.5 °C. Thermal care followed unit protocols aligned with the Neonatal Resuscitation Program (NRP) guidance for preterm infants (<32 weeks) (7). In this manuscript, DR axillary temperature denotes the neonate's axillary temperature measured in the DR, and NICU axillary temperature denotes the axillary temperature obtained on arrival to the NICU. In the delivery room, thermal management included prewarmed radiant warmers; immediate placement of infants <32 weeks' gestation in polyethylene occlusive wraps without prior drying; warmed hats and blankets; and use of prewarmed mattresses. Inspired gases were warmed and humidified whenever respiratory support was provided. As a unit policy, the ambient DR set-temperature was maintained at 23–25 °C. Transport from the DR to the NICU occurred within the same hospital via an internal corridor (approximately 200 m). Infants were transferred in a servo-controlled transport incubator; a skin-temperature probe was positioned on the anterior trunk, and the controller target range was 36.5–37.0 °C. Thermal measures initiated in the DR were maintained continuously throughout transport. On NICU admission, infants were moved to a pre-warmed servo-controlled incubator with continuation of the same target range and continuous skin-probe monitoring. The NICU axillary temperature was obtained immediately after positioning and monitor attachment. To avoid ambiguity, throughout the manuscript DR temperature refers only to the neonate's axillary temperature measured in the DR; when room ambient temperature is reported, it is explicitly described as ambient DR temperature.

### Data collection

Clinical and demographic data were extracted from the hospital's electronic medical record system (Best Care) using a structured data collection form. Maternal variables included antenatal steroid administration, hypertensive disorders, diabetes mellitus, mode of delivery, and multiple gestation. Neonatal variables included gestational age, birth weight, gender and small for gestational age status, which was defined as birth weight below the 10th percentile for gestational age. Time from birth to NICU admission was recorded in minutes.

### Outcomes

The primary (exploratory) outcome was mortality before hospital discharge. Secondary outcomes included individual major morbidities and a composite defined as the occurrence of one or more of the following: bronchopulmonary dysplasia (BPD) at 36 weeks' postmenstrual age (PMA), defined using the Jensen grading (based on level of respiratory support) (8), major IVH (Grade III or IV) (9), Retinopathy of Prematurity (ROP) requiring laser photocoagulation or anti-VEGF therapy (10), NEC stage II or higher (11), and culture-positive late-onset sepsis diagnosed after 72 h of life. Additional secondary outcomes included the duration of mechanical ventilation and total length of hospital stay.

### Study objectives

The primary objective was to compare the neonate's axillary temperature measured in the delivery room after initial stabilization (DR axillary temperature) with the axillary temperature recorded on NICU admission (NICU axillary temperature) in preterm infants <32 weeks' gestation. Secondary objectives were to evaluate agreement between DR and NICU axillary temperatures, assess the association between time to NICU admission and temperature change (NICU minus DR), and examine whether DR axillary temperature, NICU axillary temperature, and their difference are associated with mortality and major neonatal morbidities.

### Sample size and power

This retrospective cohort included all eligible infants over a three-year period; therefore, no *a priori* sample-size calculation was undertaken. Using the observed event rates and exposure prevalence, a *post hoc* power check indicated that the sample of 394 infants provided >80% power (two-sided *α* = 0.05) to detect odds ratios of ∼2.0–3.0 for key outcomes (e.g., mortality) associated with hypothermia, consistent with previously reported effect sizes (2, 12).

### Statistical analysis

Analyses were performed using IBM SPSS Statistics, Version 26.0 (IBM Corp., Armonk, NY). Distribution of continuous variables was assessed with the Shapiro–Wilk test. Because key continuous variables [delivery-room (DR) and NICU admission temperatures, time to NICU admission, duration of ventilation, and length of stay] were non-normally distributed, they are summarized as median (IQR); categorical variables as counts (%).

Paired DR and NICU temperatures for the same infant were compared using the Wilcoxon signed-rank test. Associations among DR axillary temperature, NICU axillary temperature, temperature change (NICU − DR), and time to NICU admission were explored using Spearman's rank correlation.

For descriptive group comparisons, infants were stratified into four mutually exclusive thermal-status groups: hypothermic in DR only, hypothermic in NICU only, hypothermic at both time points, and normothermic at both. Overall differences across the four groups were assessed using *χ*^2^ (or Fisher's exact) tests for categorical outcomes and Kruskal–Wallis tests for continuous outcomes. All reported *p* values are global (overall) tests across the four groups; no *post-hoc* pairwise testing was undertaken.

Time-to-event outcomes (duration of ventilation and length of stay) were analyzed with Kaplan–Meier methods and compared using the log-rank test; infants who died before extubation or discharge were censored at time of death or extubation.

To estimate independent associations, we fitted multivariable logistic regression models. For in-hospital mortality, two models were specified *a priori* with either DR hypothermia or NICU hypothermia as the exposure of interest, adjusted for gestational age, major IVH (grade III/IV), and culture-positive sepsis. For major morbidities (BPD, major IVH, NEC, PVL, ROP treatment, and mechanical ventilation), models were adjusted for gestational age. Covariates were selected for clinical plausibility and support from prior literature. Results are reported as adjusted odds ratios (aOR) with 95% confidence intervals; two-sided *p* < 0.05 was considered statistically significant.

## Results

### Study population and baseline characteristics

Three hundred ninety-four infants were born during the study period and met inclusion criteria. The median gestational age was 30.0 weeks [27.0, 31.0] and the median birth weight was 1,100 g [650, 1,500]. One third were extremely preterm (<28 weeks; 130/394, 33%) and 49% were extremely low birth weight (<1,000 g; 193/394). Males comprised 56% (256/394). Cesarean delivery occurred in 88% (345/394), 91% of mothers received antenatal corticosteroids (360/394), and 50% of infants were from multiple gestations (197/394).

Table 1 shows the early axillary temperature profile and the DR to NICU transition. Median DR and NICU temperatures were 36.7 and 36.6 °C [Δ: −0.1 °C (−0.2, 0.1)]. Hypothermia occurred in 19% in the DR and 25% at admission. Across transitions, 70.4% were normothermic at both time points, 14.2% were hypothermic at both, 10.7% converted from DR normothermia to NICU hypothermia, and 4.8% improved from DR hypothermia to NICU normothermia. The median transfer time was 16 min (13, 14); 10% had a temperature drop >0.5 °C and 4% had a rise >0.5 °C.

**Table 1:** Early axillary temperatures and transition from DR to NICU (*N* = 394).

Measure	Value		
DR axillary temperature, °C	36.7 [36.5, 36.9]		
NICU admission axillary temperature, °C	36.6 [36.4, 36.9]		
ΔTemp (NICU − DR), °C	−0.1 [−0.2, 0.1]		
Time from birth to NICU admission, min	16 [13, 20]		
Temperature drop >0.5 °C	40 (10%)		
Temperature rise >0.5 °C	16 (4%)		
DR hypothermia (<36.5 °C)	75 (19%)		
NICU hypothermia (<36.5 °C)	98 (25%)		
DR to NICU transition (counts; % of *N* = 394)			
	NICU normothermia	NICU hypothermia	Total
DR normothermia	277 (70.4%)	42 (10.7%)	319
DR hypothermia	19 (4.8%)	56 (14.2%)	75
Total	296	98	394

Hypothermia defined as axillary temperature <36.5°C. Percentages use *N* = 394 as the denominator. ΔTemp = NICU temperature minus DR temperature.

### Paired DR–NICU temperature comparison

In paired analyses of 394 infants, 179 (45.4%) had a lower temperature on NICU admission than in the delivery room, 129 (32.7%) had a higher temperature, and 86 (21.8%) were unchanged. The median paired difference (NICU − DR) was −0.1 °C [IQR: −0.2, 0.1]. A Wilcoxon signed-rank test confirmed an overall decline (*Z* = −3.132, *p* = 0.002), indicating a small but statistically significant shift toward lower temperatures during transfer.

Table 2 shows that both DR and NICU axillary temperatures were lower in infants <28 weeks and <1,000 g (all *p* < 0.001), with longer time to NICU admission in these groups (<28 weeks: 18 vs. 15 min, *p* = 0.002; <1,000 g: 18 vs. 15 m in, *p* < 0.001). The median axillary temperature change (NICU – DR) differed by gestational age (–0.1 vs. 0.0 °C, *p* = 0.031) but not by birthweight (*p* = 0.33).

**Table 2:** Temperature and time to admission by gestational Age and birth weight groups.

Variable	Total Cohort (*N* = 394)	<28 weeks	≥28 weeks	*p*-value (GA)	<1,000 g	≥1,000 g	*p*-value (BW)
Delivery room temperature (°C)	36.7 [36.5, 36.9]	36.5 [36.3, 36.7]	36.7 [36.5, 36.9]	<0.001	36.5 [36.3, 36.7]	36.7 [36.5, 37.0]	<0.001
NICU admission temperature (°C)	36.6 [36.4, 36.9]	36.5 [36.1, 36.75]	36.7 [36.5, 36.9]	<0.001	36.5 [36.2, 36.7]	36.7 [36.5, 36.9]	<0.001
Temperature difference (NICU − DR) (°C)	−0.1 [−0.2, 0.1]	−0.1 [−0.4, 0.1]	0.0 [−0.2, 0.1]	0.031	−0.1 [−0.3, 0.2]	0.0 [−0.2, 0.1]	0.330
Time to NICU admission (min)	16 [13, 20]	18 [15, 23]	15 [13, 20]	0.002	18 [14.5, 22]	15 [13, 19]	<0.001

Values are median [IQR]; axillary temperatures. *Δ*Temp = NICU − DR. *P* values from Mann–Whitney U within strata (two-sided *α* = 0.05).

**Figure 1:**
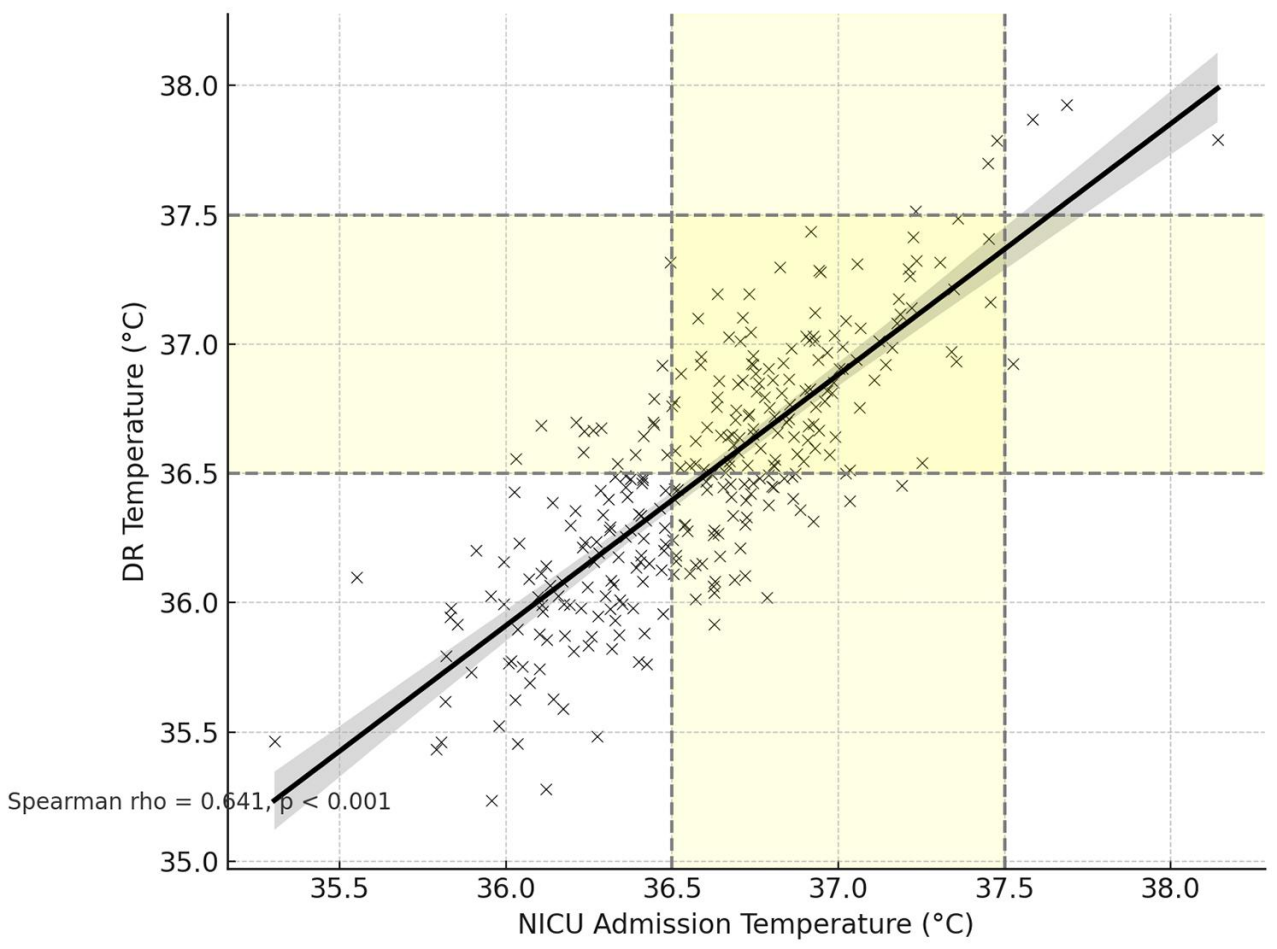
Correlation between NICU admission and delivery room temperatures with normothermia zones.

The scatter plot in Figure 1 illustrates the correlation between DR and NICU admission temperatures for individual preterm infants. Each data point represents a paired temperature measurement for a single infant. The shaded regions on both axes indicate the normothermic range, defined as 36.5 °C to 37.5 °C. A line of best fit with 95% confidence intervals is shown, demonstrating a statistically significant positive correlation between the two temperature measurements (Spearman rho = 0.641, (*p* < 0.001) (Figure 1).

### Correlations among temperature measures and transfer time

NICU admission temperature correlated positively with delivery-room (DR) temperature (Spearman rho = 0.641, *p* < 0.001). Longer time to NICU admission showed a weak association with lower NICU temperature (Spearman rho = −0.125, *p* = 0.013) but was not significantly related to DR temperature (Spearman rho = −0.081, *p* = 0.108). The temperature change (NICU − DR) was inversely related to DR temperature (Spearman rho = −0.301, *p* < 0.001), indicating larger drops when the DR temperature was higher.

Table 3 shows the association of DR and NICU hypothermia, including concurrent hypothermia in both settings, with mortality and major neonatal morbidities. Mortality was markedly higher among infants with DR hypothermia (26.3%), NICU hypothermia (22.4%), and both (25.9%) compared to normothermic infants (7.2%; *p* < 0.001). The need for mechanical ventilation followed a similar pattern, being more frequent in the DR hypothermia (78.7%), NICU hypothermia (77.6%), and both hypothermic groups (78.8%), relative to the normothermic group (55.1%; *p* < 0.001). BPD was observed in 49.0% of infants with DR hypothermia, 55.0% with NICU hypothermia, and 59.0% with both, compared to 39.0% among normothermic infants (*p* < 0.001). Major IVH was most frequent in the NICU hypothermia group (15.7%), while the DR (9.1%), both (10.8%), and normothermic (10.2%) groups showed lower rates (*p* < 0.001). PVL occurred in 10.8% of NICU hypothermic infants, compared to 8.0% (DR), 8.1% (both), and 9.1% (normothermic; *p* =  0.032) (Table 3).

**Table 3:** Association of delivery room and NICU hypothermia with mortality and Major neonatal morbidities.

Outcome	DR hypothermia (%)	NICU hypothermia (%)	Both hypothermic (%)	Normothermic (%)	Global *p*-value (*χ*^2^/Fisher)**.**
Mortality	26.3	22.4	25.9	7.2	<0.001
Mechanical ventilation	78.7	77.6	78.8	55.1	<0.001
BPD at 36 wks.	49	55	59	39	<0.001
Major IVH	9.1	15.7	10.8	10.2	<0.001
PVL	8	10.8	8.1	9.1	0.032
ROP	6.8	7.2	5.4	7.5	0.159
NEC	5.4	5.6	8	2.4	0.001
Sepsis	19.3	24.1	24.3	18.3	0.095

BPD, bronchopulmonary dysplasia; IVH, intraventricular hemorrhage; PVL, periventricular leukomalacia; ROP, retinopathy of prematurity; NEC, necrotizing enterocolitis.

*P*-values are from a single global test comparing all four groups.

Table 4 shows that, in multivariable models, higher axillary temperature at either time point was independently associated with greater odds of survival (DR: aOR: 2.37, 95% CI: 1.08–5.20, *p* = 0.031; NICU: aOR: 1.87, 95% CI: 1.04–3.36, *p* = 0.038). Gestational age was strongly favorable (per-week aOR: 1.45–1.46, both *p* < 0.001), whereas major IVH was adversely associated with survival (aOR: 2.86–3.50).

**Table 4:** Adjusted association of axillary temperature at two time-points with neonatal survival (multivariable logistic regression).

Predictor (coding)	Model A: DR axillary temperature (per 1.0 °C higher)	aOR (95% CI)	*p*-value	Model B: NICU axillary temperature (per 1.0°C higher)	aOR (95% CI)	*p*-value
Primary exposure	DR temperature	2.37 (1.08–5.20)	0.031	NICU admission temperature	1.87 (1.04–3.36)	0.038
Covariates	Gestational age (per week higher)	1.45 (1.26–1.68)	<0.001	Gestational age (per week higher)	1.46 (1.26–1.69)	<0.001
	Major IVH (yes vs. no)	3.50 (1.37–8.97)	0.009	Major IVH (yes vs. no)	2.86 (1.07–7.68)	0.037
	Culture-positive sepsis (yes vs. no)	1.11 (0.47–2.61)	0.807	Culture-positive sepsis (yes vs. no)	1.13 (0.48–2.68)	0.774

Outcome = survival to hospital discharge (aOR > 1 indicates higher odds of survival). Temperatures are axillary. DR and NICU models are fit separately, each adjusted for gestational age (per week), major intraventricular hemorrhage (grade III/IV, yes/no), and culture-positive sepsis (yes/no).

Table 5 shows that, after adjustment for gestational age, NICU hypothermia is independently associated with BPD at 36 weeks’ PMA, major IVH, and NEC (aORs: 1.89, 2.46, and 4.89, respectively), whereas DR hypothermia is associated with BPD at 36 weeks’ PMA only (aOR: 1.88).

**Table 5:** Adjusted odds ratios (aOR) for major neonatal outcomes associated with delivery-room and NICU hypothermia (models adjusted for gestational age).

Outcome (coding)	DR hypothermia aOR (95% CI)	*p* value	NICU hypothermia aOR (95% CI)	*p* value
BPD at 36 weeks’ PMA (yes/no; Jensen)	1.88 (1.02–3.46)	0.042	1.89 (1.06–3.35)	0.031
Major IVH (grade III/IV, yes/no)	1.26 (0.52–3.08)	0.614	2.46 (1.05–5.75)	0.039
NEC (stage ≥II, yes/no)	1.15 (0.32–4.13)	0.827	4.89 (1.48–16.17)	0.009
PVL (imaging-diagnosed, yes/no)	0.65 (0.20–2.13)	0.482	0.84 (0.28–2.47)	0.747
ROP requiring treatment (laser or anti-VEGF, yes/no)	1.03 (0.42–2.49)	0.953	1.40 (0.62–3.15)	0.420
Mechanical ventilation (any invasive ventilation, yes/no)	1.43 (0.71–2.88)	0.314	1.37 (0.73–2.56)	0.330

DR/NICU hypothermia defined as axillary temperature < 36.5°C at the respective time point. All models adjusted for gestational age (per week).

Table 6 shows longer unadjusted LOS and ventilation among infants with DR or NICU hypothermia by Kaplan–Meier analysis (all log-rank *p* ≤ 0.041).

**Table 6:** Length of stay and duration of ventilation by hypothermia status (kaplan–meier medians with 95% CI; global log-rank *p*-values).

Group	LOS, days median [95% CI]	*p* (log-rank)	Ventilation, days median [95% CI]	*p* (log-rank)
A. Delivery room (DR) classification				
DR hypothermia	76.0 [53.3–98.7]	<0.001	8.0 [1.8–14.2]	0.041
DR normothermia	39.0 [35.6–42.4]		6.0 [4.2–7.8]	
B. NICU admission classification				
NICU hypothermia	54.0 [43.8–64.2]	<0.001	13.0 [7.2–18.8]	0.011
NICU normothermia	38.0 [34.5–41.5]		5.0 [3.1–6.9]	

LOS, length of stay.

Log-rank *p*-values compare hypothermia vs. normothermia within each time point (DR or NICU). Estimates are unadjusted; differences may be influenced by gestational age.

## Discussion

This retrospective study of inborn preterm infants <32 weeks’ gestation examined two early time points—delivery room and NICU admission axillary temperatures—and their relationships with outcomes. We focus on thermal status at each time point and the direction of change during transfer. Despite standardized thermal care, many infants were hypothermic in the delivery room and on NICU admission, and a small net fall in temperature was common between measurements. Hypothermia at either time point was independently associated with higher mortality and major morbidities, highlighting the need for vigilant thermal management from birth through NICU admission.

In our cohort, nearly one in five infants were hypothermic after initial stabilization in the delivery room, and one in four were hypothermic upon NICU admission. These findings indicate persistent challenges in maintaining normothermia during transfer from the delivery room to the NICU. Supporting these results, a recent systematic review involving over 300,000 very preterm infants across 32 studies reported that 42% of infants were hypothermic on NICU admission (range 14%–88%) (12). Data from low- and middle-income countries further emphasize the magnitude of this issue: a meta-analysis by Beletew et al. reported a pooled hypothermia prevalence of 57.2% among hospital-based studies in East Africa (13); a study from Malawi documented a rate of 77% (15); another study from Nepal reported a rate of 64% (16); and a cross-sectional study from Sri Lanka found an incidence of 38.6% (17). In comparison, our admission hypothermia rate of 25% is lower, which may reflect differences in thermal-care protocols, institutional practices, and environments. Although our study did not evaluate ambient temperature directly, practice implications informed by existing guidelines (e.g., NRP) include attention to appropriate delivery-room ambient temperature, minimizing transfer delays, and structured team-based thermoregulation.

The median change between delivery-room and NICU axillary temperatures was −0.1 °C, with wide individual variability. Nearly half of infants had a temperature fall, and the magnitude of decline correlated inversely with time to NICU admission. This pattern suggests that longer transfers contribute to heat loss, consistent with evidence that hypothermia risk persists despite measures such as occlusive wraps and heated mattresses, particularly when transfer is prolonged (18). Although thermal blankets were used infrequently, servo-controlled incubators were used consistently and have been shown to improve temperature control and reduce deviations from normothermia (14, 19, 20).

Both delivery-room and NICU admission hypothermia were associated with higher in-hospital mortality in our cohort. In multivariate logistic regression, higher axillary temperature at each time point remained independently associated with greater odds of survival after adjustment for gestational age, major IVH (grade III/IV), and culture-positive sepsis, consistent with the higher crude mortality observed among hypothermic infants. These findings align with large multicenter cohorts: a European study of 5,697 infants <32 weeks reported that admission temperature <35.5 °C doubled early mortality (RR: 2.41, 95% CI: 1.45–4.00) (21); the Chinese Neonatal Network (5,913 very preterm infants) found adjusted odds of mortality of 1.41 for 36.0–36.4 °C and 1.93 for <35.5 °C (22); and a multicenter study of 1,247 VLBW infants showed a more than fourfold increase with moderate/severe hypothermia (23). Beyond the immediate hospitalization, lower admission temperature has also been linked with increased risk of death or moderate-to-severe neurodevelopmental impairment at 3 years in very low-birth-weight infants (24).

Our data showed that NICU admission hypothermia—more consistently than delivery-room hypothermia—was independently associated with adverse outcomes, including BPD, major IVH, and NEC after adjustment for gestational age. This pattern is concordant with large cohort reports e.g., Lyu et al. (2); Mohamed (6), and a recent systematic review encompassing over 300,000 infants (12). It also complements work showing that persistent hypothermia during transition to the NICU is linked to higher risks of PVL, BPD, and mortality in infants <33 weeks’ gestation (25). Our analysis adds paired DR and NICU temperatures within the same cohort and uses GA-adjusted models to examine outcome associations.

The direction and magnitude of the temperature change between the delivery room and NICU likely reflect the quality of immediate postnatal stabilization and transfer. In our data, NICU admission hypothermia showed stronger associations with IVH and NEC than delivery-room hypothermia, consistent with the concept that sustained or progressive heat loss exerts greater physiologic stress than a brief early deviation. Prolonged hypothermia is linked to impaired cerebral autoregulation and reduced splanchnic perfusion—mechanisms relevant to IVH and NEC—and may also contribute to pulmonary morbidity. Cohort studies report higher odds of BPD and adverse respiratory outcomes when admission temperature is low (5, 26). Biologically, hypothermia can blunt surfactant production, lower lung compliance, increase pulmonary vascular resistance, and raise metabolic oxygen demand, thereby predisposing to ventilator dependence. In our cohort, hypothermic infants required mechanical ventilation more often. Taken together, these observations support interpreting temperature at NICU arrival as a clinically meaningful marker of risk, rather than proposing specific management recommendations beyond the scope of our analysis.

Extending these associations from morbidity to care utilization, we examined time-dependent outcomes. In Kaplan–Meier analyses, infants who were hypothermic in the delivery room or at NICU admission had longer time to discharge and longer duration of mechanical ventilation than normothermic peers. These are unadjusted survival comparisons; because gestational age strongly influences both outcomes and differs across temperature groups, the results should be interpreted as associations rather than causal effects.

A key strength of this study is a well-defined single-centre cohort with consistently documented paired axillary temperatures at two clinically relevant time points: immediately after delivery-room stabilization and on NICU admission, under standardized thermal protocols. The cohort size allowed descriptive comparisons by broad gestational-age groups (<28 vs. ≥28 weeks), though it was not powered for week-by-week analyses. Nevertheless, important limitations remain. The retrospective design precludes causal inference and may introduce selection bias. The exact timing of measurements relative to birth and any delays in documentation could not be fully standardized. Data on ambient room temperatures and transport conditions were incomplete, and information on team composition and training was not available, leaving room for residual confounding. Finally, this is a single-centre study, which may limit generalizability to settings with different environments, resources, or thermal care practices.

## Conclusion

In this single centre cohort of infants <32 weeks’ gestation, hypothermia was common both after delivery room stabilization and on NICU admission, and temperature often declined during transfer. After adjustment for gestational age, lower temperature at either time point was associated with higher in hospital mortality. NICU admission hypothermia was additionally linked to major morbidities, including BPD, major IVH, and NEC. These findings indicate that thermal management may need attention at two junctures, immediately after birth and during transport and early admission, because the current approach did not consistently prevent hypothermia. Future work should test targeted strategies that shorten transfer time and strengthen the application of existing thermal measures across the entire transition.

## Data Availability

The raw data supporting the conclusions of this article will be made available by the authors, without undue reservation.
